# Distribution of sialic acid receptors and experimental infections with different subtypes of influenza A viruses in Qinghai-Tibet plateau wild pika

**DOI:** 10.1186/s12985-015-0290-8

**Published:** 2015-04-14

**Authors:** Yan Li, Haixia Xiao, Chaobin Huang, Haigang Sun, Laixing Li, Jingliang Su, Juncai Ma, Di Liu, Han Wang, Wenjun Liu, George F Gao, Xiangdong Li, Jinghua Yan

**Affiliations:** CAS Key Laboratory of Pathogenic Microbiology and Immunology, Institute of Microbiology, Chinese Academy of Sciences, Beijing, 100101 China; Laboratory of Protein Engineering and Vaccines, Tianjin Institute of Industrial Biotechnology, Chinese Academy of Sciences, Tianjin, 300308 China; State Key Laboratory of the Agro-Biotechnology, College of Biological Sciences, China Agricultural University, Beijing, 100193 China; College of Veterinary Medicine, China Agricultural University, Beijing, 100193 China; Northwest Institute of Plateau Biology, Chinese Academy of Sciences, Xining, 810008 China; Network Information Center, Institute of Microbiology, Chinese Academy of Sciences, Beijing, 100101 China; Research Network of Immunity and Health, Beijing Institutes of Life Science, Chinese Academy of Sciences, Beijing, 100101 China; Office of Director-General, Chinese Center for Disease Control and Prevention, Beijing, 102206 China

**Keywords:** Sialic acid receptors, Experimental infection, Influenza A viruses, Wild plateau pika, Pathogenic changes

## Abstract

**Background:**

The plateau pika (*Ochotona curzoniae*) is a small rabbit-like mammal that lives at high altitudes in the Qinghai-Tibet plateau and is in close contact with birds. Following the outbreak of highly pathogenic avian influenza (HPAI) H5N1 during 2005 in the migratory birds of Qinghai Lake, two clades of H5N1 have been found in pikas. However, the influenza virus receptor distribution in different tissues of this animal and its susceptibility to influenza A viruses have remained unclear.

**Methods:**

The sialic acid receptor distribution tropism in pika was investigated using fluorescent *Sambucus nigra* and biotinylated *Maackia amurensis* I and II. Furthermore, the replication of three influenza A viruses H1N1, H3N2, and H5N1 in this animal was examined by immunohistochemistry and RT-PCR. Morphological and histopathological changes caused by infection were also analyzed with hematoxylin and eosin (H & E) staining.

**Results:**

Human influenza virus-recognizing SAα2,6Gal receptors are widely expressed in the lung, kidney, liver, spleen, duodenum, ileum, rectum, and heart, whereas avian influenza virus-recognizing SAα2,3Gal receptors are strongly expressed in the trachea and lung of pika. M1 could be detected in the lungs of pikas infected with H1N1, H3N2, and H5N1 by either immunostaining or RT-PCR, and in the brain of H5N1-infected pikas. Additionally, three subtypes of influenza A viruses were able to infect pika and caused varying degrees of pneumonia with epithelial desquamation and alveolar inflammatory cell infiltration. Slight pathological changes were observed in H1N1-infected lungs. A few small bronchi and terminal bronchioles were infiltrated by lymphocytic cells in H3N2-infected lungs. In contrast, serious lung damage, such as alveolar capillary hyperemia, edema, alveolar collapse, and lymphocytic infiltrations was observed in H5N1-infected group. Furthermore, neural system changes were present in the brains of H5N1-infected pikas.

**Conclusions:**

SAα2,6Gal receptors are extensively present in many of the tissues and organs in wild plateau pika, whereas SA2,3Gal-linked receptors are dominant on the tracheal epithelial cells. H1N1, H3N2, and H5N1 were able to infect pika and caused different degrees of pathogenic changes in the lungs. Altogether, these results suggest that wild pika has the potential to be a host for different subtypes of influenza A viruses.

**Electronic supplementary material:**

The online version of this article (doi:10.1186/s12985-015-0290-8) contains supplementary material, which is available to authorized users.

## Background

Influenza A viruses are important pathogens in the poultry industry and also pose a large threat to public health. Sixteen hemagglutinin (HA) subtypes and nine neuraminidase (NA) subtypes of influenza A viruses are found in waterfowl [1], which are considered the natural reservoir of influenza A viruses. Moreover, two novel bat-derived subtypes of influenza A viruses, H17N10 and H18N11, were recently discovered respectively in little yellow-shouldered bats (*Sturnira lilium*, family *Phyllostomidae*) and flat-faced fruit bats (*Artibeus planirostris*) by sequencing analyses [2-4]. However, as yet, those two novel influenza viruses lack classical functions for the viral envelope proteins, the HA cannot bind to any traditional influenza virus receptors, and NA loses the neuraminidase activity to remove sialic acid [3-8]. The discovery of influenza-like viruses in bats has highlighted the concerns of the scientific community over a possible broader host range of the virus.

As stated above, migratory and wild birds (in particular, waterfowl) are generally considered to be the natural hosts for all known subtypes of influenza A viruses, with some subtypes being more adapted to infect humans, birds, pigs, horses, and other animals. Certain subtypes of influenza A viruses that have adapted to infect humans are currently circulating among the human population worldwide, *e.g.*, the H1N1 and H3N2 viruses. Whereas some other HA subtypes of avian influenza virus, such as H5, H6, H7, H9, and H10, have crossed the interspecies barrier and have acquired the ability to infect humans but lack the ability to circulate among humans. Human infections with such avian influenza viruses have ranged from mild symptoms (*e.g.*, conjunctivitis and influenza-like illness) to severe disease, including deaths [9-18]. Worryingly, HPAI virus H5N1 and the low pathogenic avian influenza (LPAI) A virus H7N9 have caused a great number of human infections and deaths. According to the records from the World Health Organization (WHO), H5N1 influenza has caused 650 human infections, with 386 confirmed deaths, from to 2003 to January 24, 2014 [19]. The novel H7N9 virus that emerged in March 2013 has caused 419 infections and 127 deaths until July, 2014 [20].

Waterfowl and shorebirds have historically been considered the primary natural reservoir for influenza viruses, allowing their circulation with low pathogenicity among the flock. However, the outbreak of H5N1HPAI at Qinghai Lake astonished the world with the death of over 6,000 migratory birds in 2005 [21,22]. Since 2005, H5N1 has spread, following the migration routes of migratory birds, to Europe and some parts of Africa, and has caused severe poultry outbreaks and human infections [23]. In addition, H5N1 has been identified in the plateau pikas [24], which are found at the Qinghai-Tibet plateau and share their local environment with a plethora of wild birds. Consequently, concerns have been raised as to whether pika is a novel natural host of H5N1 and/or other subtypes of influenza viruses.

A major determinant of infection is the presence of sialosaccharide receptors on the host cell surface to which viral HA can bind. Avian influenza viruses preferentially bind to sialic acid (SA) receptors that are linked to galactose by an α 2,3 linkage (SAα2,3Gal), while human and classical swine viruses show preference for receptors with an α 2,6 linkage (SAα2,6Gal) [25,26]. Distribution tropisms of receptors among different hosts could account for variation in their susceptibility to infection by influenza viruses. The distribution of the influenza virus receptors SAα2,3Gal and SAα2,6Gal has been assessed in several avian and mammal species, and the distribution patterns vary among different species [26-35]. As an important member of the Qinghai-Tibet plateau eco-system, plateau pika was found to be the host of H5N1 influenza viruses [24]. However, little is known about influenza virus receptor distribution in this animal and the pathogenicity caused by H5N1 infection. Thus, we explored the influenza virus receptor distribution in different organs of pika and investigated the susceptibility of the plateau pika to a variety of subtypes of influenza A viruses (including H1N1, H3N2, and H5N1) in this study.

## Results

### Distribution of SAα2,3Gal and SAα2,6Gal receptors in the different organs of pika

To evaluate the potential role of receptor distribution in the susceptibility of pika to influenza virus infection, we investigated the distributions of SAα2,3Gal and SAα2,6Gal receptors in various organs, such as the trachea, lung, duodenum, ileum, rectum, heart, liver, kidney, brain, and spleen, by using biotinylated *Maackia amurensis* (MAA) lectin (red) I (MAAI) and II (MAAII) for SAα2,3Gal binding and FITC-labeled *Sambucus nigra* (SNA) lectin (green) for SAα2,6Gal binding. To eliminate the non-specific staining of MAAI, MAAII and SNA lectins, we used human trachea exclusively expressing SAα2,6Gal receptor and duck duodenum with only SAα2,3Gal expression to determine the background levels of lectin staining. It was clear that these three lectins displayed very low background staining (Additional file 1: Figure S1).

**Figure 1:**
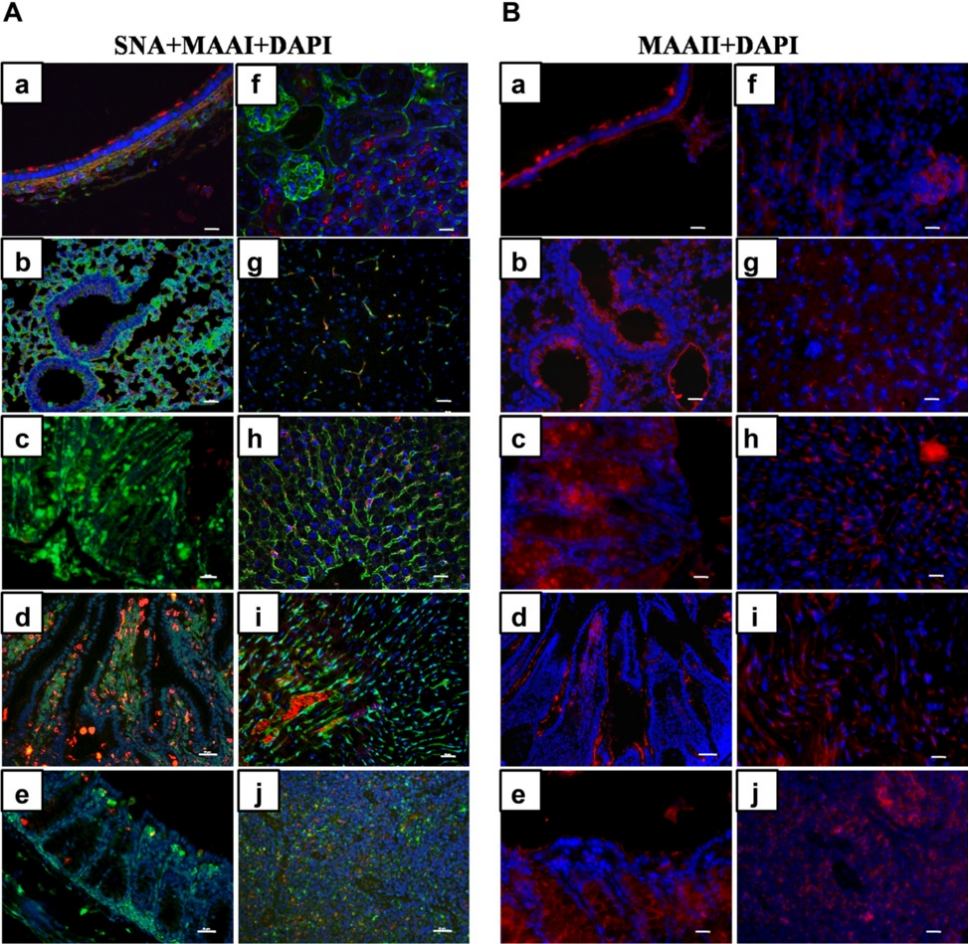
Selective presence of SAα2,3Gal (MAAI, MAAII lectin) and SAα2,6Gal (SNA lectin) receptors in different pika organs. (A) Expression of SAα2,3Gal (red) and SAα2,6Gal (green) receptors was detected by MAAI and SNA lectin staining in contrast to nuclear staining (blue) with DAPI and presented as composite confocal images in many pika organs including the trachea **(a)**, lung **(b),** duodenum **(c)**, ileum **(d)**, rectum **(e)**, kidney **(f),** brain **(g)**, liver **(h)**, heart **(i)**, and spleen **(j)** as indicated above. Bar, 100 μm. (B) Expression of SAα2, 3Gal (red) receptors was revealed by MAAII staining relative to nuclear staining (blue) with DAPI. The organs are labeled as in Figure 1A. Bar, 100 μm.

Based on this result, we performed lectin staining in the different tissues and organs of pika. Individual staining with DAPI, SNA, MAAI, and MAAII for different tissues was shown in (Additional file 2: Figure S2). And those overlapping images for different staining combinations were clearly presented in Figure 1. We observed strong red (MAAI and MAAII) and weak green staining at the tip of the ciliated columnar epithelium, suggesting that SAα2,3Gal receptors rather than SAα2,6Gal are widespread on the tracheal epithelial cells (Figure 1A-a and B-a). We also found that the tracheal lamina propria and mucous glands express both SAα2,3Gal and SAα2,6Gal linked receptors when MAAI, MAAII, and SNA were used for staining (Figure 1A-a and B-a). However, alveolar cells are rich in SAα2,6Gal receptors and also have a strong staining with MAAII lectin but have a faint MAAI signal (Figure 1A-a and B-a). In the lower respiratory tract, alveoli cells display predominant expression of SAα2, 6Gal receptors. Contrary to the findings in chickens (*Gallus gallusdomesticus*) and ducks (*Anas*) [28], very few epithelial cells were positive for either MAAI or SNA staining in the smaller and terminal bronchiole of pika (Figure 1A-b). In contrast to MAAI, MAAII lectin was abundant in the smaller and terminal bronchiole of this animal (Figure 1B-b). The fluorescent staining along the epithelial lining of the mucosa demonstrated the presence of SAα2,6Gal in the goblet cells and epithelium of the duodenum, ileum, rectum, and lacteals (Figure 1A-c, A-d, and A-e). In contrast, SAα2,3Gal receptors stained with MAAI lectin showed significant expression in the lacteals, but MAAI exhibited weak staining in both the goblet cells and ciliated epithelial cells (Figure 1A-c, A-d, and A-e). Strong MAAII lectin staining was mainly observed on the apical side of the ciliated epithelial cells (Figure 1B-c, B-d, and B-e).

The expression patterns of avian and human receptors were also defined in the kidney, as kidney cell lines are usually utilized for influenza viruses’ isolation and propagation. The MAAI, MAAII, and SNA staining revealed that both avian and human receptors could be detected in the kidney of pika, but MAAII and SNA demonstrated much stronger staining in the epithelial cells from the glomeruli and proximal tubules. In contrast, SAα2,3Gal rather than SAα2,6Gal receptors were present in the epithelial cells from the distal tubules (Figure 1A-f and B-f).

In agreement with the findings in mouse (*Mus musculus*) [29], both SAα2,3Gal and SAα2,6Gal receptors were rare in the brain cells from pika (Figure 1A-g and B-g). In the liver, the SAα2,6Gal and SAα2,3Gal stained with MAAII lectin were predominant on the hepatocytes and sinusoid, while weak SAα2,3Gal signal stained with MAAI was found in the liver sinusoid, as well as on the membrane of Kupffer cells (Figure 1A-h and B-h).

In the heart, MAAI, MAAII and SNA displayed very strong staining in the cardiac myocytes (Figure 1A-i and B-i), suggesting that both α2,6Gal and α2,3Gal are widely expressed in the heart. The dominant expression of both avian and human receptors makes heart a very susceptible target for avian and human influenza viruses.

In the spleen, MAAI, MAAII, and SNA staining was detected in both the red and white pulps, but the SAα2,6Gal signal was much more intense. A similar distribution pattern was also found in the lymphatic sheath around the artery in the white pulp regions (Figure 1A-j and B-j).

### Pika could be infected with H1, H3, and H5 three subtypes of influenza A viruses

After ensuring that the animals were not H1, H3, or H5 sero-positive with hemagglutination inhibition (HI) assay, 118 pikas (100-170 g) were divided into four groups. Three groups were intranasally inoculated with 7 × 10^4^ pfu of H1, H3, or H5 influenza virus, and the fourth group was inoculated with PBS as a control. At five days post infection, most influenza virus-infected pikas, except 10 animals infected with H5N1, did not display characteristic signs of illness, such as shivering, ruffled fur, and hunched posture. To investigate the infection of three different influenza viruses, we tested the viral M RNA in the lungs at 5 days post infection (d.p.i.) using RT-PCR. The three different influenza viruses-challenged groups (H1N1, H3N2, and H5N1) presented different infectivities (30%, 21.4%, and 43.3%, respectively) (Table 1).

**Table 1:** **Detection of viruses among the infected pikas with RT-PCR analysis**

**Virus strains**	**No. of pika**	**PCR positive**	**Infectivity (%)**
A/WSN/1933	30	9	30.0
A/Jiangxi/262/2005	28	6	21.4
A/great black-headed gull/Qinghai/1/2009	30	13	43.3
PBS	30	0	0

**Figure 2:**
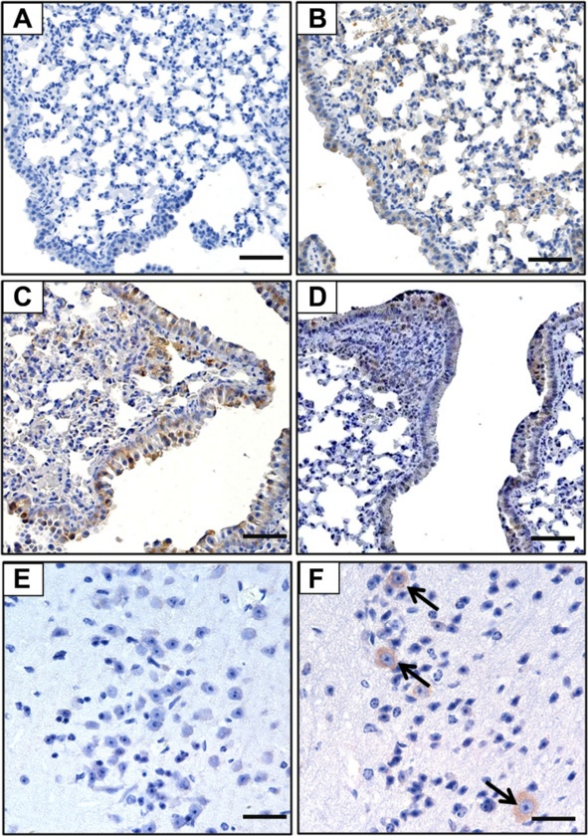
Detection of viral antigen M1 protein in the lung of H1N1- and H3N2-infected pikas, as well as in the lung and brain from H5N1-infected pikas. Immunohistochemical analyses of viral antigen distribution in the tissues of pika using a monoclonal antibody against M1 protein in **(A)** Uninfected lung, **(B)** H1N1-infected lung, **(C)** H5N1-infected lung, **(D)** H3N2-infected lung, **(E)** Uninfected brain, and **(F)** H5N1-infected brain. Arrows denote the M1-positive Purkinje cells. M1 is expressed on the membrane and in the cytoplasm of epithelial cells of the small bronchi, terminal bronchioles, and alveolar cells. Bar, 100 μm.

To confirm the virus infection and viral antigen distribution in the tissues of the infected animals, immunohistochemical analyses with monoclonal antibody against M1 were performed. The expression of viral antigen M1 could be detected in the lung tissues of H1N1-, H5N1-, and H3N2-infected pikas, which were confirmed by the RT-PCR assay. The M1 viral antigen could be found in both ciliated and alveolar epithelial cells all over the lung in both the H1N1- (Figure 2B) and H5N1-infected pikas (Figure 2C), while M1 antigen could only be detected focally in the epithelial cells of a few bronchi and alveolar cells in the H3N2-infected animals (Figure 2D). Additionally, influenza virus antigen staining revealed that M1 antigens were mainly observed in the Purkinje cells, some of the largest neurons that coordinate voluntary movements in the brain of H5N1-infected pikas (Figure 2F). Taken together, pika could be infected by different subtypes of influenza A viruses at high doses, though infectivity and infection tropism in the lungs were various.

### Pathological changes in the organs and tissues of influenza viruses-infected pika

**Figure 3:**
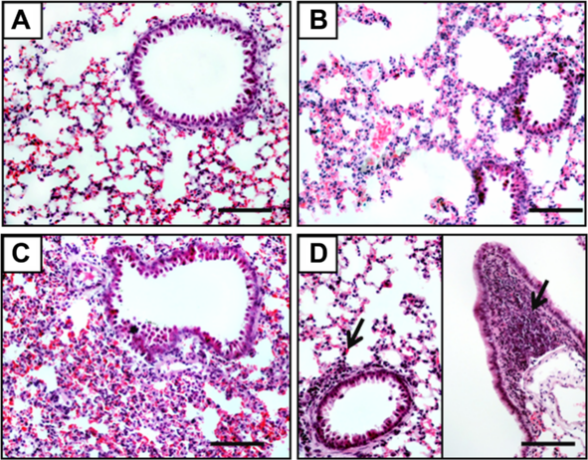
Histopathological analyses of lungs from different influenza virus-infected or uninfected pikas by H & E staining. **(A)** The uninfected lung serves as the normal control (n = 5, control group; n = 5, infected group). **(B)** Slight alveolar capillary congestion, edema, and thickening of the interstitium of alveolar cells with local lymphocyte infiltration were observed in H1N1-infected lung; no exudation within the alveolar lumen was found in the H1N1-infected lung. **(C)** Alveolar capillary hyperemia, edema, and alveolar collapse with lymphocyte infiltration were found throughout of the H5N1-infected lung. **(D)** The alveolar structure was normal, with only a few small bronchi and terminal bronchioles with lymphocytic infiltration in the H3N2-infected lung (arrows). Bar, 100 μm.

To elucidate any pathological damage that may occur in influenza A viruses-infected pikas, various tissues, including the lung, spleen, kidney, brain, liver, intestine, and trachea, were collected from pikas infected with avian H5N1, human H1N1, and H3N2 influenza viruses and stained with H & E. We observed various levels of severity in the virus-infected lungs (Figure 3). Compared to the PBS control group (Figure 3A), the alveolar structure appeared normal, and a few small bronchi and terminal bronchioles were infiltrated with lymphocytic cells in the H3N2-infected lung tissue (Figure 3D). Slight pathological changes, such as alveolar capillary congestion, edema, and thickening of the alveolar interstitium with local lymphocytic infiltrations, were observed in the H1N1-infected lungs. Even though the alveolar cavity became smaller, no exudation within the alveolar space was found (Figure 3B). In contrast, serious damage, including a large area of alveolar capillary hyperemia, edema, alveolar collapse, and lymphocytic infiltrations, was present in the lungs of H5N1-infected pika (Figure 3C).

**Figure 4:**
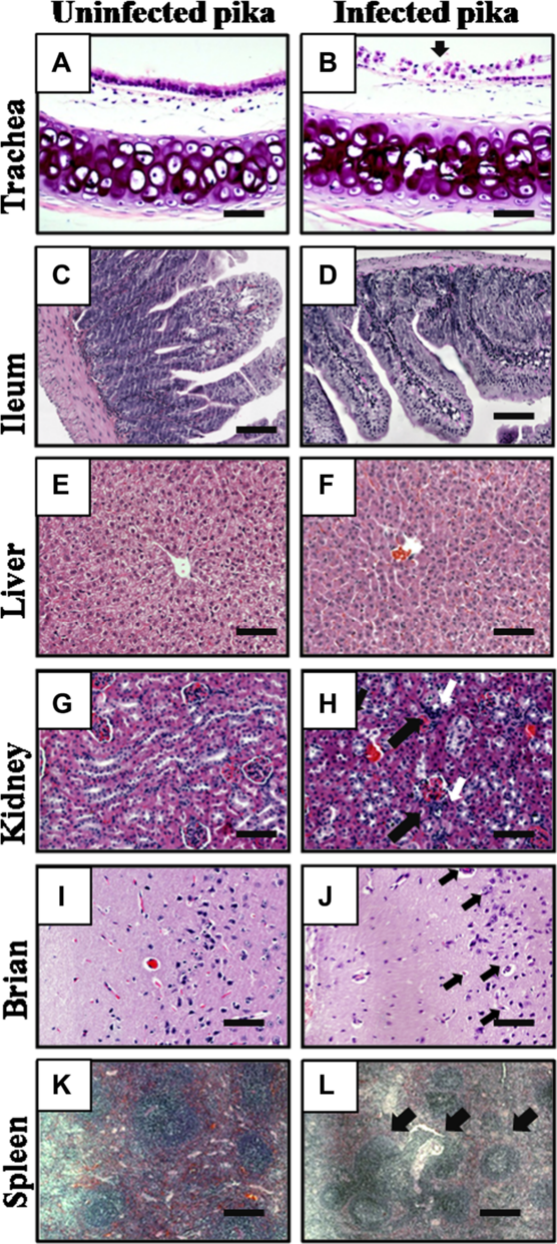
Histopathology in different tissues of H5N1-infected pika. **(A, C, E, G, I, K)**: Representative specimen from the uninfected control group (n = 5, control group; n = 5 per infected group). **(B, D, F, H, J, L)**: Representative specimen from the H5N1-infected group. **(A)** Uninfected Trachea. **(B)** Desquamation of the apoptotic tracheal epithelial cells was observed in the virus-infected trachea (arrow). **(C)** Uninfected ileum. **(D)** No notable abnormalities were observed in the ileum from the infected pika. **(E)** Uninfected liver. **(F)** No significant abnormalities were observed in the liver from the infected pika. **(G)** Uninfected kidney. **(H)** Lymphocytic infiltrations around glomerular afferent arterioles in the kidney (arrows). **(I)** Uninfected cerebellum. **(J)** Apoptotic Purkinje cells were observed in the infected cerebellum (arrows). **(K)** Uninfected spleen. **(L)** Enlarged white pulps were observed in the virus-infected spleen (arrows). Bar, 50 μm.

Unlike the severity observed in the lung tissue, ileum and livers from H5N1-infected pikas showed no significant alterations (Figure 4D, F). Similarly, obvious pathogenic changes were not found in other organs, including the trachea, kidney, liver, ileum, brain, and spleen in the H1N1- and H3N2-infected pikas (data not shown). Notably, increases in the desquamation of apoptotic ciliated epithelial cells in tracheas (Figure 4B), lymphocytic infiltrations around glomerular afferent arterioles in the kidney (Figure 4H), apoptotic Purkinje cells in the cerebellum (Figure 4J), and expansion of the white pulp in the spleen (Figure 4L) were obvious in the H5N1-infected pikas. Apoptotic progress observed in Purkinje neurons is the result of H5N1 infection and replication, which was demonstrated by immunostaining for the M1 antigen. The pathological changes were also the cause of the signs of illness in pika. These results demonstrate that a high-dose infection of influenza A viruses is able to induce variant viral pneumonia and multi-organ abnormalities in wild pika.

## Discussion

Following the outbreak of severe H5N1 HPAI in wild birds [21,22], plateau pika infections with two distinct lineages, a mixed Vietnam H5N1 virus lineage and a wild bird Qinghai-like H5N1 virus lineage, were reported in 2007 around Qinghai Lake [24]. Considering that wild birds share the same habitats with pika and that some small birds often enter into pika caves for feeding, critical questions were raised concerning whether influenza virus is highly pathogenic to pika and whether pika could act as an influenza virus-carrier. To answer these questions, we first investigated the influenza virus receptor distribution in plateau pika. One interesting finding was that SAα2,6Gal linked saccharide receptors are present in high amounts in all of the tested organs and tissues, especially in the lungs. The wide presence of SAα2,6Gal linked receptors may facilitate the replication of the human origin influenza A viruses in pika. It also suggests that pika could be more important as an intermediate host for the generation of influenza viruses with increased binding affinity to SAα2,6Gal and SAα2,3Gal receptors.

We also demonstrated that pika could be experimentally infected with various subtypes of influenza A viruses, *e.g.*, human viruses H1N1, H3N2, and the avian virus H5N1. However, virus replication in the lung tissue and viral infectivity differed among these three subtypes, which is consistent with the predominant receptor expression in the trachea and lung and the receptor preferences of human H1N1 and H3N2 and avian H5N1. Dominant expression of SAα2,3Gal in both the trachea and lung facilitates the infection and replication of H5N1 avian influenza virus. In contrast, SAα2,6Gal was distributed mainly in the lungs rather than in the trachea. This explains why much more viral antigen M1-positive cells were stained in the lung of avian receptor-preferring H5N1-infected pikas, and why a higher infection rate occurred in the avian H5N1-challenged group than those in the human receptor-preferring H1N1 and H3N2 infected ones.

Furthermore, H1N1, H3N2, and H5N1 caused different types of damage in the lungs of infected pikas. Similar to the H5N1-infected rabbits (*Oryctolagus cuniculus*) [24], H1N1-and H3N2-infected pikas displayed mild pathological changes with lymphocytic infiltrations in the lung. In contrast, serious damage, such as alveolar capillary hyperemia, edema, alveolar collapse, and lymphocytic infiltrations, were observed in the H5N1-infected group. Different degrees of lung damage were correlated with the number of viral antigen M1-positive cells, as well as viral infectivity.

In contrast to the lung tissue, the presence of avian receptor SAα2,3Gal in the trachea, lung, kidney, and spleen may facilitate H5N1 attachment, which in turn caused the lesions and lymphocytic infiltrations in these tissues. We also demonstrated that avian H5N1 infected the pika central neuron system (CNS) by attacking Purkinje neurons in the cerebellum, which is in consistent with H5N1 infection in ferret (*Mustela putorius furo*) and crow (*Corvus*) [36,37]. These results demonstrate that pika is more susceptible to H5N1 avian influenza virus, and the pathogenic response of pika to influenza viruses is subtype- or strain-dependent, though the mechanism involved needs to be further explored.

Taken together, we demonstrated that pika is capable of being an influenza host, though human origin H1N1 and H3N2 viruses had relative low replication efficiency in the lung than avian H5N1. As the influenza virus continues to circulate within the pika population, the influenza viruses that have been found in wild plateau pika are most likely transmitted from wild birds. The presence of SAα2,6Gal and SAα2,3Gal receptors in many organs suggest that pika could act as an efficient virus reservoir and possible mixing vessels. Consequently, we would advise that monitoring of viruses among the wild bird populations in the Qinghai-Tibet plateau should also be extended to monitor the wild pika.

## Methods

### Virus strains and virus amplification

Influenza viruses A/WSN/1933 (H1N1) and A/Jiangxi/262/2005 (H3N2) were amplified in MDCK cells; A/great black-headed gull/Qinghai/1/2009 (H5N1) was propagated in 10 day-old SPF embryonated eggs. Viral titers were determined by plaque assays. Briefly, confluent MDCK cells in 12-well plates were washed twice with PBS and then infected with 500 μl virus in DMEM (GIBCO, Grand Island, NY) at a concentration range from 10^−1^ to 10^−6^ for 1 hour (h) at 37°C with gentle shaking. The inocula were then removed, and the cells were washed twice and overlaid with DMEM containing 1% agarose (Promega, Madison, WI) and 5 μg/ml TPCK treated trypsin. After overlays are solidified, the cells were incubated at 37°C with 5% CO_2_ for 72 hours (hrs). Cells were then fixed with 4% formaldehyde in PBS for 1 h and stained with neutral red at room temperature (RT). All experiments with the H5N1 virus were performed in a biosafety level 3 laboratory.

### Lectin detection of SAα2,3Gal and SAα2,6Gal

Sections of various tissues were deparaffinized and rehydrated as described below for H & E staining. After being washed in distilled water for 5 minutes at RT, sections were heat-treated with the antigen-retrieval solution (citrate buffer, pH 6.0) four times, each time for 4 minutes. Before being stained with lectins, sections were blocked with 10% horse serum (GIBCO, Grand Island, NY) for 10 minutes at RT. Tissue sections were incubated with specific FITC-labeled SNA lectin to detect SAα2,6Gal receptor (Vector Laboratories, Burlingame, CA) and biotinylated MAAI and MAAII lectins for SAα2,3Gal (Vector Laboratories, USA ) for 2 hrs at 37°C. After being washed with PBS three times, the sections were incubated with PE-conjugated streptavidin (Sigma, Santa Clara, CA) for 2 hrs at RT. The sections were washed in PBS three times to remove unbound streptavidin and then counterstained with 4,6-diamidino-2-phenylindole (DAPI) (Roche, Switzerland).

### Animal infection with influenza A viruses

The pika animals in this experiment were caught in regions around Haibei Alpine Grassland Ecosystem Research Station in Qinghai Province, China. Sera collected from pikas via the lateral tail veins were tested by HI and neutralization assays with H1, H3, and H5 influenza viruses to rule out influenza virus sero-positive animals. Female adult pikas with body masses of approximately 100-170 g were selected and kept in captivity for 7 days to adapt to the environment before being infected with influenza viruses. Food and water were provided every day. The animals were anesthetized with pentobarbital sodium salt (Sigma, 40 mg/kg) via intraperitoneal injection and intranasally infected with 7.0 × 10^4^ pfu of H5N1, H1N1, or H3N2 virus. Five days post virus infection, pikas were sacrificed, and tissues, including the heart, liver, spleen, half of the lung, trachea, kidney, brain, and intestine, were collected and stored in neutral formalin; the other half of the lungs were frozen in liquid nitrogen for RNA extraction.

The Ethics Committee for animal experimentation of the Institute of Microbiology, Chinese Academy of Sciences, approved all of the animal experiments (Registry No.CASPMI2008001).

### Immunohistochemistry (IHC) detection of influenza A virus M1 protein

Immunohistochemical staining was performed as described below. Reagents used in this study were described as follows: mouse anti-M1 monoclonal antibody (1:200 dilution, a gift from Professor Wenjun Liu, Institute of Microbiology, CAS.), biotinylated goat-anti-mouse secondary antibody (1:200 dilution, Vector Laboratories, USA), and strepavidin-conjugated HRP (1:200 dilution, Jackson Immuno Research, West Grove, PA).

### Viral RNA extraction and RT-PCR

To identify infection in experimental pikas, viral RNA from the lung of each pika was extracted as described in the RNeasy Mini kit (Qiagen, Hilden, Germany). Briefly, 30 mg lung tissue was disrupted and homogenized in 600 μl RLT buffer supplied in the kit, and the nucleic acids were eluted with 30 μl nuclease-free water. Lungs from uninfected pikas were used as the negative control. Viral cDNA was immediately obtained using a Reverse Transcription System kit (Promega, Madison, WI) with primer Uni12 5′-AGCAAAAGCAGG-3′. Amplification of the M segment was performed with primers UMF 5′-TATTCGTCTC AGGGAGCAAAAGCAGGTAG-3′, and UMR5′-ATATCGTCTCGTATTAGTAGAAACAAGGTAGTTTTT-3′.

### Morphological and histological analyses

Tissues collected from pikas were dehydrated with increasing concentrations of ethanol, vitrified by dimethylbenzene, and then embedded in paraffin. After being embedded, the tissues were sectioned with a rotary microtome (Hestion ERM3000, China). Sections (5 μm-thicknesses) were deparaffinized in dimethylbenzene, rehydrated using graded ethanol, and stained with H & E for histopathological examination. Before being mounted, sections were dehydrated and vitrified. Mounted slides were observed under a fluorescence microscope (IX71 Olympus Co., Ltd., Tokyo) and photographed with a Nikon DXM1200F (Nikon Co., Tokyo).

## Additional files

Additional file 1: Figure S1. Expression of SAα2,3Gal (MAAI, MAAII lectin) and SAα2,6Gal (SNA lectin) receptors in duck duodenum and human trachea. **(A)** Expression of SAα2,3Gal (red) and SAα2,6Gal (green) receptors were detected by MAAI and SNA lectins staining in the contrast to nuclear staining (blue) with DAPI, and presented with composite confocal images in human trachea and duck duodenum. Bar, 200 μm. **(B)** Expression of SAα2,3Gal (red) receptors were revealed by MAAII staining in the distinguish with nuclear staining (blue) with DAPI. Different organs labeling is the same order with those in supplementary Figure 1A. Bar, 200 μm. SAα2,3Gal receptors stained with MAAI and MAAII lectins are exclusively present in the duck duodenum, whereas, human trachea only shows the expression of SAα2,6Gal receptors. Additional file 2: Figure S2. Selective presence of SAα2,3Gal (MAAI, MAAII lectin) and SAα2,6Gal (SNA lectin) receptors in different organs of pika. Expression of SAα2,3Gal (MAAI, MAAII, red) and SAα2,6Gal (SNA, green) receptors were detected by MAAI, MAAII and SNA lectins staining in the contrast to nuclear staining (blue) with DAPI, and presented with composite confocal images in many pika organs including **(A)** trachea, lung, duodenum, ileum, rectum, and others **(B)** kidney, brain, liver, heart and spleen indicated above. Bar, 100 μm. SAα2,3Gal (MAAI, MAAII lectin) and SAα2,6Gal (SNA lectin) receptors are selectively present in these organs. SAα2,6Gal receptors are widely expressed in the lung, kidney, liver, spleen, duodenum, ileum, rectum and heart, whereas SAα2,3Gal receptors are dominant in the trachea, lung, ileum, kidney, liver, heart and spleen.
