# Supplementary figures and images for: Distribution of sialic acid receptors and experimental infections with different subtypes of influenza A viruses in Qinghai-Tibet plateau wild pika
Source: Virol J. 2015 Apr 14;12:63. doi: 10.1186/s12985-015-0290-8 (PMC4409991; doi:10.1186/s12985-015-0290-8)

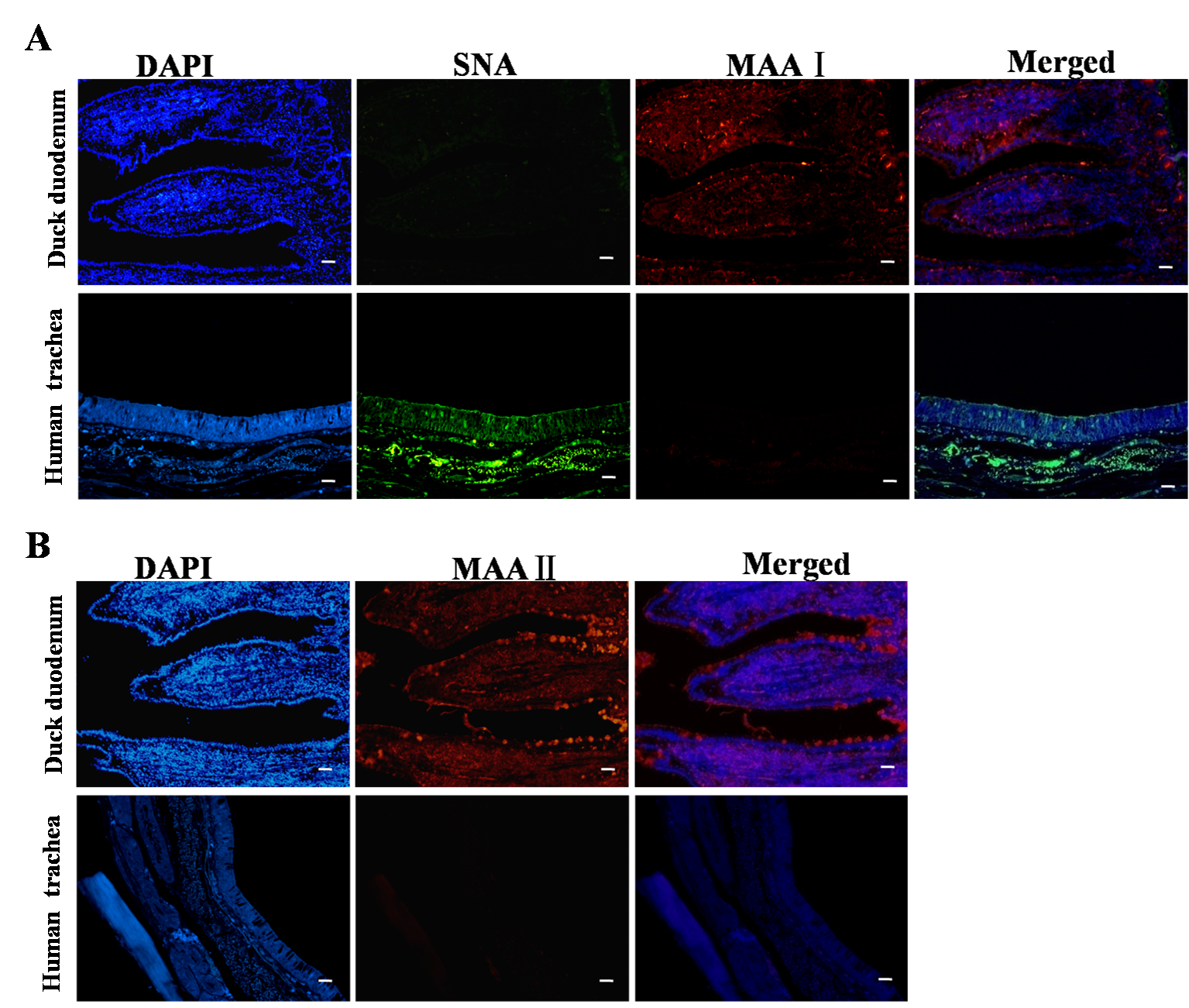

Supplement: Additional file 1: Figure S1. — Expression of SAα2,3Gal (MAAI, MAAII lectin) and SAα2,6Gal (SNA lectin) receptors in duck duodenum and human trachea. (A) Expression of SAα2,3Gal (red) and SAα2,6Gal (green) receptors were detected by MAAI and SNA lectins staining in the contrast to nuclear staining (blue) with DAPI, and presented with composite confocal images in human trachea and duck duodenum. Bar, 200 μm. (B) Expression of SAα2,3Gal (red) receptors were revealed by MAAII staining in the distinguish with nuclear staining (blue) with DAPI. Different organs labeling is the same order with those in supplementary Figure 1A. Bar, 200 μm. SAα2,3Gal receptors stained with MAAI and MAAII lectins are exclusively present in the duck duodenum, whereas, human trachea only shows the expression of SAα2,6Gal receptors. [file 12985_2015_290_MOESM1_ESM.tiff]

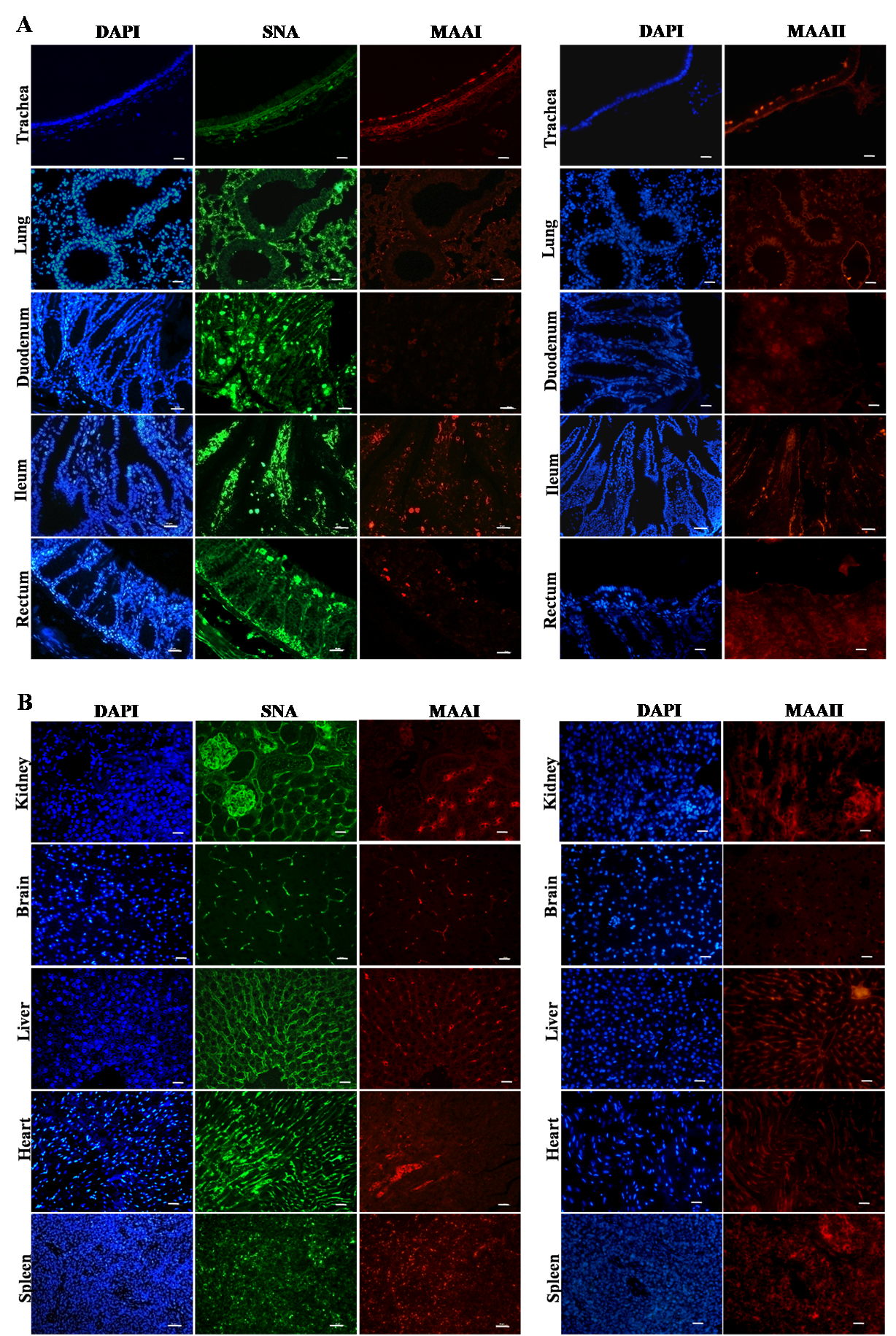

Supplement: Additional file 2: Figure S2. — Selective presence of SAα2,3Gal (MAAI, MAAII lectin) and SAα2,6Gal (SNA lectin) receptors in different organs of pika. Expression of SAα2,3Gal (MAAI, MAAII, red) and SAα2,6Gal (SNA, green) receptors were detected by MAAI, MAAII and SNA lectins staining in the contrast to nuclear staining (blue) with DAPI, and presented with composite confocal images in many pika organs including (A) trachea, lung, duodenum, ileum, rectum, and others (B) kidney, brain, liver, heart and spleen indicated above. Bar, 100 μm. SAα2,3Gal (MAAI, MAAII lectin) and SAα2,6Gal (SNA lectin) receptors are selectively present in these organs. SAα2,6Gal receptors are widely expressed in the lung, kidney, liver, spleen, duodenum, ileum, rectum and heart, whereas SAα2,3Gal receptors are dominant in the trachea, lung, ileum, kidney, liver, heart and spleen. [file 12985_2015_290_MOESM2_ESM.tiff]
